# *Trypanosoma cruzi* Adjuvants Potentiate T Cell-Mediated Immunity Induced by a NY-ESO-1 Based Antitumor Vaccine

**DOI:** 10.1371/journal.pone.0036245

**Published:** 2012-05-02

**Authors:** Caroline Junqueira, Ana Tereza Guerrero, Bruno Galvão-Filho, Warrison A. Andrade, Ana Paula C. Salgado, Thiago M. Cunha, Catherine Ropert, Marco Antônio Campos, Marcus L. O. Penido, Lúcia Mendonça-Previato, José Oswaldo Previato, Gerd Ritter, Fernando Q. Cunha, Ricardo T. Gazzinelli

**Affiliations:** 1 Laboratório de Imunopatologia, Centro de Pesquisas René Rachou, Fundação Oswaldo Cruz, Belo Horizonte, Brazil; 2 Departamento de Bioquímica e Imunologia, Universidade Federal de Minas Gerais, Belo Horizonte, Brazil; 3 Instituto Cerrado Pantanal, Fundação Oswaldo Cruz, Campo Grande, Brazil; 4 Division of Infectious Diseases and Immunology, University of Massachusetts Medical School, Worcester, Massachusetts, United States of America; 5 Departamento de Farmacologia, Faculdade de Medicina de Ribeirão Preto, Universidade de São Paulo, Ribeirão Preto, Brazil; 6 Instituto de Biofísica Carlos Chagas Filho, Centro de Ciências da Saúde, Universidade Federal do Rio de Janeiro, Rio de Janeiro, Brazil; 7 Ludwig Institute for Cancer Research, New York Branch at Memorial Sloan–Kettering Cancer Center, New York, New York, United States of America; Federal University of São Paulo, Brazil

## Abstract

Immunological adjuvants that induce T cell-mediate immunity (TCMI) with the least side effects are needed for the development of human vaccines. Glycoinositolphospholipids (GIPL) and CpGs oligodeoxynucleotides (CpG ODNs) derived from the protozoa parasite *Trypanosoma cruzi* induce potent pro-inflammatory reaction through activation of Toll-Like Receptor (TLR)4 and TLR9, respectively. Here, using mouse models, we tested the *T. cruzi* derived TLR agonists as immunological adjuvants in an antitumor vaccine. For comparison, we used well-established TLR agonists, such as the bacterial derived monophosphoryl lipid A (MPL), lipopeptide (Pam3Cys), and CpG ODN. All tested TLR agonists were comparable to induce antibody responses, whereas significant differences were noticed in their ability to elicit CD4^+^ T and CD8^+^ T cell responses. In particular, both GIPLs (GTH, and GY) and CpG ODNs (B344, B297 and B128) derived from *T. cruzi* elicited interferon-gamma (IFN-γ) production by CD4^+^ T cells. On the other hand, the parasite derived CpG ODNs, but not GIPLs, elicited a potent IFN-γ response by CD8^+^ T lymphocytes. The side effects were also evaluated by local pain (hypernociception). The intensity of hypernociception induced by vaccination was alleviated by administration of an analgesic drug without affecting protective immunity. Finally, the level of protective immunity against the NY-ESO-1 expressing melanoma was associated with the magnitude of both CD4^+^ T and CD8^+^ T cell responses elicited by a specific immunological adjuvant.

## Introduction

NY-ESO-1 is a human cancer/testis antigen that is frequently expressed in a variety of cancer cells, but not in normal adult tissues apart from testis [1], [2]. Both humoral and T cell-mediated immunity (TCMI) specific for NY-ESO-1 develop in patients with NY-ESO-1-positive tumors; and several major histocompatibility complex (MHC) class II and I restricted peptides have been defined as the epitopes recognized by CD4^+^ T as well as CD8^+^ T lymphocytes, respectively [3], [4], [5]. The immunogenicity and tissue distribution indicate that NY-ESO-1 is an excellent candidate antigen for prophylactic and therapeutic anticancer vaccines. Hence, different vaccine formulations employing NY-ESO-1 have been developed aiming at efficient antitumor activity. Most formulations combine heterologous prime-boost protocols to achieve satisfactory immunogenicity and tumor regression in experimental models [6], [7]. Importantly, different clinical trials have shown the ability of NY-ESO-1 vaccines to induce specific cytolytic T lymphocytes as well as CD4^+^ T cell-mediated immune responses in humans [8], [9].

However, the quality of the T cell response and protection against tumors still remains a major challenge for vaccine development. One of the main difficulties is the limited availability of licenced immunological adjuvants that induce strong and long-lasting TCMI with the least undesirable effect. The discovery that activation of Toll-Like Receptors (TLRs) promote the initiation and development of both T cell and B cell responses has intensified the search for new immunological adjuvants [10]. Indeed, various microbial components as well as synthetic components previously shown to work as immunological adjuvants were proven to be TLR agonists [11]. When exposed to microbial components, cells from the innate immune system, synthesize high levels of pro-inflammatory cytokines and express co-receptors, in order to initiate the activation process of naïve T cells, bridging the innate and acquired immunity [12]. Importantly, dendritic cells (DCs) activated with TLR agonists will produce interleukin (IL)-12 and influence the differentiation of CD4^+^ T cells into the T helper type 1 (Th1) phenotype, which orchestrate the establishment of cell-mediated immunity as well as the production of interferon-gamma (IFN-γ)-inducible Ig isotypes that are often involved in host resistance to tumors [13], [14], [15]. Furthermore, activation of antigen presenting cells favors cross-presentation, allowing presentation of exogenous antigens via MHC class I [16], [17]. Currently, several vaccines based on association of tumor antigens with defined TLR agonists (*e.g.*, Poly I:C, Monophosphoryl Lipid A, Flagellin, CpG oligodeoxynucleotides, Imiquimods) are being tested in pre-clinical and clinical trials [15], [18], [19].

We have previously shown that glycosylphosphatidylinositol (GPI) anchors linked to mucin-like glycoproteins, and the ceramide-containing GPI anchors, also termed glycoinositolphospholipids (GIPL), present in outer plasmatic membrane of the *Trypanosoma cruzi* are immunostimulatory molecules for TLR2 and TLR4, respectively [20], [21]. It was also demonstrated that CpG oligodeoxynucleotide (CpG ODN) motifs derived from the *T. cruzi* genome activate TLR9 [22]. We believe that this is the molecular basis of the highly polarized Th1 response and strong TCMI elicited during infection with *T. cruzi* parasites.

In this study, we evaluated the *T. cruzi* derived TLR agonists as immunological adjuvants in vaccine formulations employing ovalbumin (OVA) or NY-ESO-1 as antigens. Our results show that formulations containing either CpG ODNs or GIPL induced immune responses mediated by CD4^+^ Th1 lymphocytes. In particular, parasite derived CpG ODNs, but not GIPL, elicited a potent IFN-γ response by CD8^+^ T lymphocytes. We also evaluated adjuvant-induced hypernociception and showed that there was no correlation with the quality of the immune response, and alum was the main cause of “pain” in the vaccine formulations. Immune-mediated protection against melanoma development directly correlated with the magnitude of IFN-γ responses by both NY-ESO-1-specific CD4^+^ T as well as CD8^+^ T cells. Finally, the use of the analgesic Paracetamol (PCM) did not alter the immunogenicity and protective immunity elicited by these novel vaccine formulations employing parasite adjuvants.

## Materials and Methods

### Ethics Statement

Mice experiments were approved by and conducted according to animal welfare guidelines of the Ethics Committee of Animal Experimentation from Universidade Federal de Minas Gerais under the title *“Parasite derived adjuvants for cancer vaccines”* and approved protocol number 19/2008.

### Mice and cell lines

C57BL/6 mice, originally obtained from Jackson Laboratory, were kept under standard pathogen-free conditions. Six- to eight-week-old females, weight-matched, were used in the different experimental groups. CHO transfected cells [23] and B16F10 wild type (WT) and NY-ESO-1-transfected [24] cell lines were maintained in culture in RPMI 1640 supplemented with 100 IU/ml penicillin, 10 µg/ml streptomycin, 10% heat-inactivated fetal bovine serum (FBS), 1 µg/ml tylosin and maintained at 37°C in a 5% CO_2_ incubator. B16F10 cell line expressing NY-ESO-1 were supplemented with 300 µg/ml geneticin.

### Removal of lipopolysaccharides from ovalbumin

Chicken OVA (Sigma-Aldrich, St. Louis, MO) was diluted in pyrogen-free saline at 10 mg/ml and depleted of the endotoxin activity using five cycles of Triton X-114 extractions [25]. OVA concentration was determined by Bradford assay, and adjusted to 1 mg/ml. The endotoxin levels in purified OVA were measured by Gel-Clot *Limulus amoebocyte* lysate reagent (Charles River Laboratories, Wilmington, MA) and found to be below the limit of detection (0.03 EU/ml).

### NY-ESO-1: recombinant protein and T cell epitope mapping

The recombinant NY-ESO-1 protein was produced under Good Manufacturing Practice (GMP) at the Ludwig Institute for Cancer Research/Cornell University Partnership Production Facility in Ithaca, New York.

*In silico* prediction of MHC class I (H2-Kb, H2-Db and H2-Lb) and class II (H2-IAb) ligands from NY-ESO-1 were determined by the software Bimas [26] and SYFPEITHI [27], respectively. The MHC restricted peptides from Ovalbumin (OVA CD4^+^ T cell epitope – ISQAVAAHAEINEAGR and OVA CD8^+^ T cell epitope – SIINFEKL) and the restricted peptides from NY-ESO-1 (NY-ESO-1 CD4^+^ T cell epitopes - CD4-1 QAEGRGTGGSTGNAN, CD4-2 AGGPGEAGATGGRGP, and CD4-3 FYLAMPFATPMEAEL; as well as NY-ESO-1 CD8^+^ T cell epitopes - CD8-1 TVSGNILTI, CD8-2 SCLQQLSLL, and CD8-3 LLEFYLAM) were synthesized by standard N-9-fluorenylmethyloxycarbonyl on a PSSM8 multispecific peptide synthesizer (Shimadzu, Kyoto, Japan) by solid- phase synthesis with a scale of 30 µM and a purity >85%, as determined by reverse-phase HPLC. Their identities were confirmed by Autoflex III Maldi-TOF/TOF Mass Spectrometer (Bruker Daltonics, Billerica, MA). For in vitro lymphocytes restimulation, each peptide was used at 10 µM final concentration.

### *T. cruzi* GIPLs: purification and *in vitro* immunostimulation

The isolation and purification of GIPL has been previously described in detail [23], [28]. Briefly, epimastigotes of *T. cruzi* (Y and Tulahuen strains) were grown in BHI-hemin medium supplemented with 5% FBS. *T. cruzi* in stationary growth phase were extracted three times with cold water and the remaining cell pellet extracted with 45% aqueous phenol. The aqueous layer from the phenol extraction was dialyzed and applied to a column of Bio-Gel P-100 (Bio-Rad, Hercules, CA). The excluded material was lyophilized and the free GPIs extracted by chloroform/methanol/water (10∶10∶3). The virtual absence of contaminating peptidic material was confirmed by the absence of peptide-derived signals in nuclear magnetic resonance spectroscopy and mass spectrometry analyses of the purified material. The GIPL preparation tested negative for Lipopolysaccharides (LPS) content using a Limulus amebocyte lysate test, with a limit of detection of 0.03 EU/ml (Charles River Laboratories, Wilmington, MA).

The CHO reporter cell lines (CHO/CD14, expressing endogenous functional TLR4; 7.19/CD14/TLR2, expressing TLR2; and the 7.19 clone, expressing neither TLR2 nor functional TLR4) were generated as described [23]. These cell lines contain a human CD25 gene reporter under the control of E-selectin promoter and CD25 expression is completely dependent upon NF-kB translocation. Macrophage-activating lipopeptide 2 kDa (MALP-2; Alexis Biochemicals, San Diego, CA) and LPS (Sigma-Aldrich, St. Louis, MO) were used as controls at 10 ηg/ml and 200 ηg/ml, respectively. TLR4^+^ cells that showed activation were additionally treated 15 min with 1 µg/ml of polymyxin B (Sigma-Aldrich, St. Louis, MO), an inhibitor of LPS, prior to GIPLs exposure. Cells were exposed to the different molecules, and analyzed 18 hours after stimulation, through staining with PE CD25 (Caltag Laboratories, Burlingame, CA) and flow cytometry analyzes by BD CellQuest Pro Software (Becton, Dickinson and Company, Franklin Lakes, NJ). The results were obtained by subtracting the percentage of cells expressing the reporter gene in stimulated versus not stimulated cell populations. Ten thousand cells were analyzed in each sample.

### CpG ODN: sequences and *in vitro* immunostimulation

Table 1 shows the sequences of mouse B-class-like CpG ODNs, mouse-human hybrid B class-like CpG ODNs and human B-class-like CpG ODNs derived from *T. cruzi* genome [22]. *T. cruzi* derived CpG ODNs, as well as positive and negative controls for GpG ODNs were synthesized by Alpha DNA (Montreal, Quebec, Canada) as phosphorothioate ODNs and purified by oligonucleotide purification cartridge.

**Table 1:** Sequences of synthesized CpG oligonucleotide.

Name	Sequence
B-class-616	TCGACGTTTGGATCGAT
B-class-344*	TCGACGTTTGGATCGGT
B-class-297*	TCCTCGTTTTGACGTG
B-class-377	TCGTCGTTGTCGTC
B-class-181	TTGTCGTTGTCGTT
B-class-145	TTGTCGTCGTCGTT
B-class-138A	TTGTCGTAGTCGTCGTT
B-class-138B	TCGTCGTCGTCGTT
B-class-130	TCGTCGTTGTCGTT
B-class-128*	TCGTCGTTGTAGTCGTA
B-class-126	TCGTCGTGGTCGTT
B-class-114	TCGTCGTAGTCGTT
B-class-110	TCGTCGTTGTCGT
B-class-1891	TCGTCGCTCTCCTCGTC
B-class-1400	TCGTCGGTGGCGTCGCT
B-class-599	TCGTCGTCGTCGTCGTC
B-class-338	TGGTCGTCGTGGTCGTC
B-class-278	TCATCGTTGTGCTCGTT
B-class-190	GCGTCGAATTGTCGTT
B-class-220	TCATCGTTATGCTCGTT
CpG 7909 [CpG(+)]*	TCGTCGTTTTGTCGTTTTGTCGTT
GpG [CpG (−)]	TCCAGGACTTCTCTCAGGTT
CpG 2007	TCGTCGT TGTCGTTTTGTCGTT

* CpG ODNs used for immunization protocolsz.

For IL-12 production assays, inflammatory macrophages from mice injected with 1,5 ml of 3% thioglycolate were plated at 5×10^6^ cells/ml, and incubated at 37°C and 5% CO_2_ for 72 h in the presence or absence of LPS or CpG ODNs at different concentrations. IL-12 concentrations were determined in cell culture supernatants with DuoSet ELISA (R&D Systems, Minneapolis, MN). Two hundred thousand peripheral blood mononuclear cells (PBMCs) were cultured in 96-well plates in the presence of CpG ODNs at different concentrations associated with DOTAP (Roche, Indianapolis, IN) for 24 h, and interferon-alpha (IFN-α) was measured in the cell culture supernatant with DuoSet ELISA (R&D Systems, Minneapolis, MN).

### Vaccine formulations and immunization protocols

Vaccine formulations were prepared with 10 mg/ml OVA or 5 mg/ml NY-ESO-1 and TLR agonists co-adsorbed in 30% (v/v) of alum Rehydragel L.V. solution (Reheis, Berkeley Heights, NJ) for 1 hour at room temperature in a tube rotator. After incubation, saline solution was added to each sample to the final concentration of 100 µg/ml of the antigen. The final concentration of each agonist was used as follows: 10 µg/ml Synthetic MPLA (InvivoGen, San Diego, CA); 10 µg/ml Pam3Cys (Alexis Biochemicals, San Diego, CA); 180 µg/ml CpG [positive control (+), negative control (−), or *T. cruzi* derived B344, B297, B128]; 500 µg/ml GIPL (TcGY or TcGTH). All the procedures were developed using endotoxin free supplies in a sterile environment.

Four to six mice per group were immunized with alum, alum plus OVA or alum plus OVA plus TLR agonists. Each mouse received three subcutaneous (s.c.) doses of vaccine formulations 14 days apart. Sera were collected 9 days after the last immunization and spleens were collected 12 days later for analysis of immune responses. Mice that were immunized with NY-ESO-1 received only two immunizations 21 days apart. In parallel, a group of mice were treated with 10 mg/kg of PCM by the oral route 30 minutes prior to each immunization dose.

### Measurement of antibody and T cell responses

Vaccinated mice were bled from the retro-orbital plexus under ether anesthesia. Antigen-specific antibodies were measured in sera from immunized mice by enzyme-linked immunosorbent assay (ELISA). Secondary Ab, peroxidase-conjugated goat anti-mouse total Immunoglobulin G (IgG), IgG1 or IgG2c (SouthernBiotech, Birmingham, AL) were used and the reactions were detected with 3,3′,5,5′-tetramethylbenzidine reagent (Sigma-Aldrich, St. Louis, MO).

For IFN-γ production assays, splenocytes from vaccinated mice were prepared in complete RPMI supplemented with 100 U/ml rIL-2 (R&D Systems, Minneapolis, MN), plated at 5×10^6^ cells/ml and incubated at 37°C and 5% CO_2_ for 72 h in the presence or absence of epitopes derived from OVA or NY-ESO-1 proteins. IFN-γ concentrations were determined in cell culture supernatants with DuoSet ELISA (R&D Systems, Minneapolis, MN). To confirm peptides specificity, CD8^+^ T and CD4^+^ T cells were isolated from total splenocytes using Dynabeads (Invitrogen Dynal, Oslo, Norway), and plated at 5×10^6^ cells/ml for peptides addition. Total splenocyte from vaccinated mice were stained *ex vivo* with FITC CD3 (BD Pharmingen, San Jose, CA) and PE-Cy5 CD8 (BD Pharmingen, San Jose, CA) antibodies as well as PE H2-Db CD8 NY-ESO-1_127–135_ tetramer and analyzed in a FACSCalibur (Becton, Dickinson and Company, Franklin Lakes, NJ).

### Evaluation of mechanical hypernociception

The term hypernociception rather than hyperalgesia or allodynia is used to define the decrease in nociceptive withdrawal threshold [29]. Mechanical hypernociception was tested in mice as previously reported [30]. In a quiet room, mice were placed in acrylic cages (12×10×17 cm) with wire grid floors, 15–30 min before the start of testing. The test consisted of evoking a hindpaw flexion reflex with a handheld force transducer (electronic anesthesiometer; IITC Life Science, Woodland Hills, CA) adapted with a 0.5-mm^2^ polypropylene tip. The investigator was trained to apply the tip perpendicularly to the central area of the plantar hindpaw with a gradual increase in pressure. The gradual increase in pressure was manually performed in blinded experiments. The upper limit pressure was 15 g. The end-point was characterized by the removal of the paw followed by clear flinching movements. After paw withdrawal, the intensity of the pressure was automatically recorded, and the final value for the response was obtained by averaging three measurements. The animals were tested before and after treatments. The results are expressed by the delta (▵) withdrawal threshold (in g) calculated by subtracting the zero time mean measurements from the mean measurements at the indicated times after drug or solvent (controls) injections. Withdrawal threshold was 9.0±0.2 g (mean±S.E.M.) before injection of solvent or hypernociceptive agents.

### B16F10 or B16 NY-ESO-1 melanoma challenge

Mice vaccinated with recombinant NY-ESO-1 were challenged s.c. at day 21 after boost with 5×10^4^ B16F10 melanoma cells expressing or not the cancer antigen NY-ESO-1. Tumor growth was monitored during 40 days.

### Statistical analysis

All the statistic analysis were performed by GraphPad Prism Software Version 5.0 b (GraphPad Software, Inc., La Jolla, CA). The non-parametric group comparison was developed by Mann-Whitney test and the parametric data by T test. The tumor development data was analyzed by two-way ANOVA with additional Bonferroni post-test analysis. Survival curves were analyzed by Log-rank test. Differences were considered significant when p<0.05.

## Results

### *T. cruzi* derived GIPLs activate TLR4 and promote antigen-specific IgG2c and CD4^+^ T cell responses

**Figure 1:**
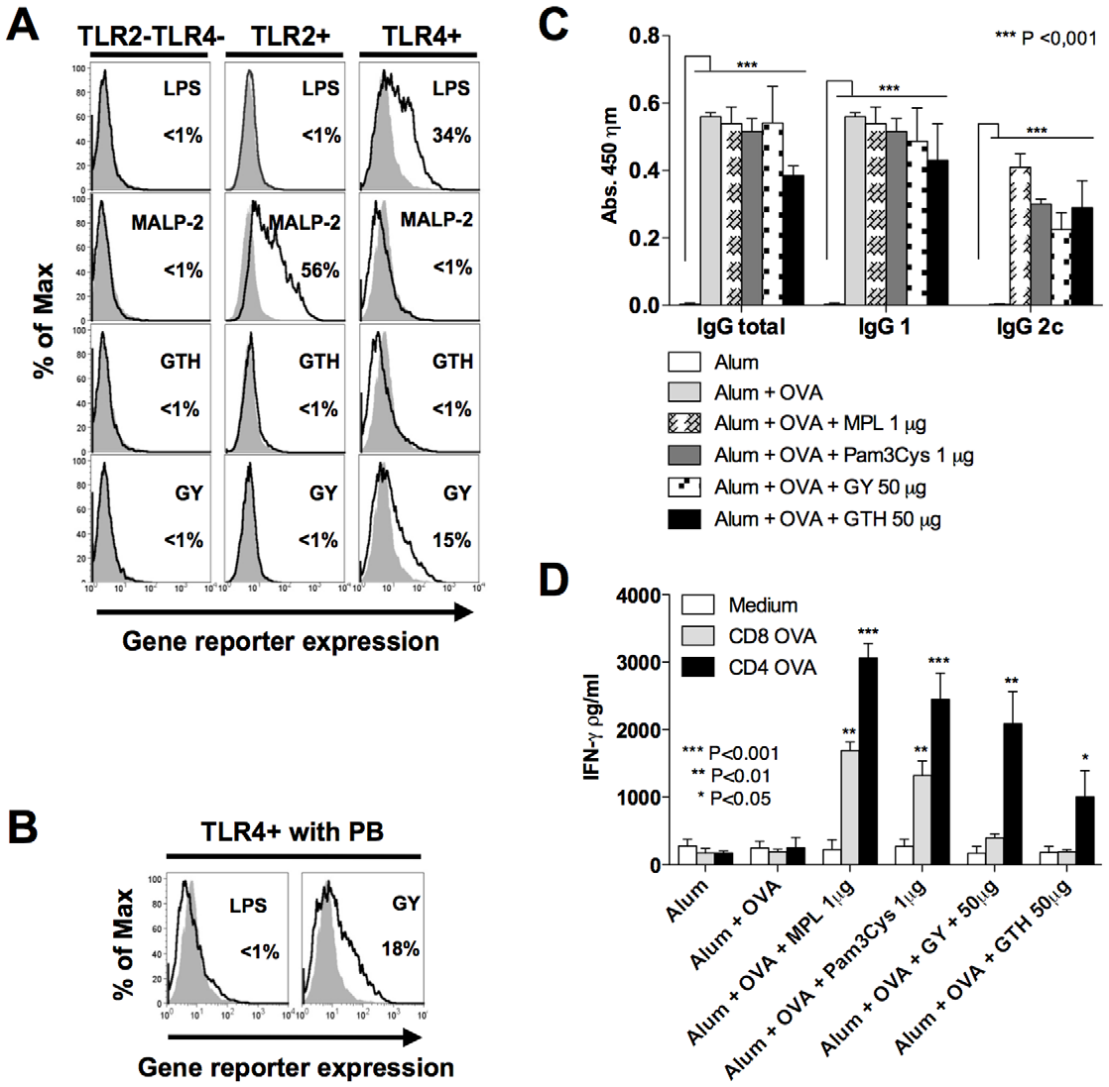
*T. cruzi* derived GIPLs are TLR4 agonists and promote high levels of antigen-specific IgG2c antibodies as well as IFN-γ production by CD4^+^ T cells. (**A**) CHO cells control (TLR2^−^/TLR4^−^) or expressing TLR2 (TLR2^+^) or TLR4 (TLR4^+^) were either left untreated (solid gray) or exposed to 100 µg/ml of GIPLs from *Trypanosoma cruzi* Tulahuen (GTH), Y strain (GY) (black line). MALP-2 (10 ηg/ml) and LPS (200 ηg/ml) were used as positive controls for activation of TLR2^+^ or TLR4^+^, respectively. (**B**) TLR4^+^ cells were activated with different preparations of GIPLs in the presence of polymyxin B (PB). LPS was used as control. (**C**) OVA specific immune responses induced by immunization with TLR2 or TLR4 agonists associated with OVA absorbed in alum. Mice were immunized with three doses on days 0, 14 and 28. The production of total IgG, IgG1 and IgG2c were assessed by ELISA using the sera from immunized mice, at day 9 after the second boost. (**D**) To assess the levels of IFN-γ production by T lymphocytes from vaccinated mice, splenocytes were collected 21 days after the third immunization dose and stimulated with either CD4^+^ T or CD8^+^ T cell epitopes from OVA. The results are representative of two independent experiments yielding similar results. Asterisks indicate that differences were statistically significant, when comparing T cell response from mice receiving different vaccine formulations.

The ability of GIPLs to activate TLR2 and TLR4 was investigated in CHO cells functional for TLR4 (TLR4^+^) or not functional for TLR4 and stably transfected with TLR2 (TLR2^+^) (Figure 1). As positive controls, we used LPS and MALP-2 as TLR4 and TLR2 agonists, respectively. CHO cells transfected only with CD25 reporter gene were used as negative controls (TLR2^−^TLR4^−^). In this system, expression of CD25 is completely dependent upon NF-kB translocation. In Figure 1A it is shown that GIPLs derived from the Y strain of *T. cruzi* (GY) leads to 15% enhancement of CD25 expression in TLR4^+^ cells. To certify that activation of TLR4^+^ cells were not due to LPS contamination, the same experiments were performed in the presence of polymyxin B (PB), a compound that is known to bind LPS and prevent TLR4 activation. The experiment shown in Figure 1B demonstrates activation of TLR4^+^ cells by *T. cruzi* GIPL even after treatment with PB. In contrast activation with LPS was completely blocked by pre-treatment with PB. Thus, we consider that the activation of TLR4^+^ CHO cells by GIPLs from the Y strain of *T. cruzi* was not due to a LPS contamination.

The GIPLs were also compared with well-established TLR2 and TLR4 agonists, Pam3Cys and MPL, respectively, for their capacity to promote immunological responses in an immunization protocol using OVA as antigen. All the evaluated TLR agonists associated with alum plus OVA induced antigen specific total IgG, as well as IgG1 and IgG2c. In contrast, mice that received only alum plus OVA induced antigen specific total IgG and IgG1, but not IgG2c isotype (Figure 1C). Splenocytes from vaccinated mice were restimulated with OVA-specific peptides for CD4^+^ T and CD8^+^ T cells. The levels of IFN-γ in the supernatant of splenocyte cultures was measure by ELISA (Figure 1D). Our data shows that MPL and Pam3Cys promoted IFN-γ production by OVA-specific CD4^+^ T and CD8^+^ T cells. In contrast, the GIPL from *T. cruzi* induces IFN-γ production only by CD4^+^ T cells. Since the GIPL derived from the Y strain of *T. cruzi* (GY) was the best parasite adjuvant to induce CD4^+^ T cell responses, it was chosen to be used in the vaccine formulations employing NY-ESO-1 as antigen.

### CpG ODNs derived from the *T. cruzi* genome activate TLR9 and promote antigen specific IgG2c as well as CD4^+^ T and CD8^+^ T cell responses

**Figure 2:**
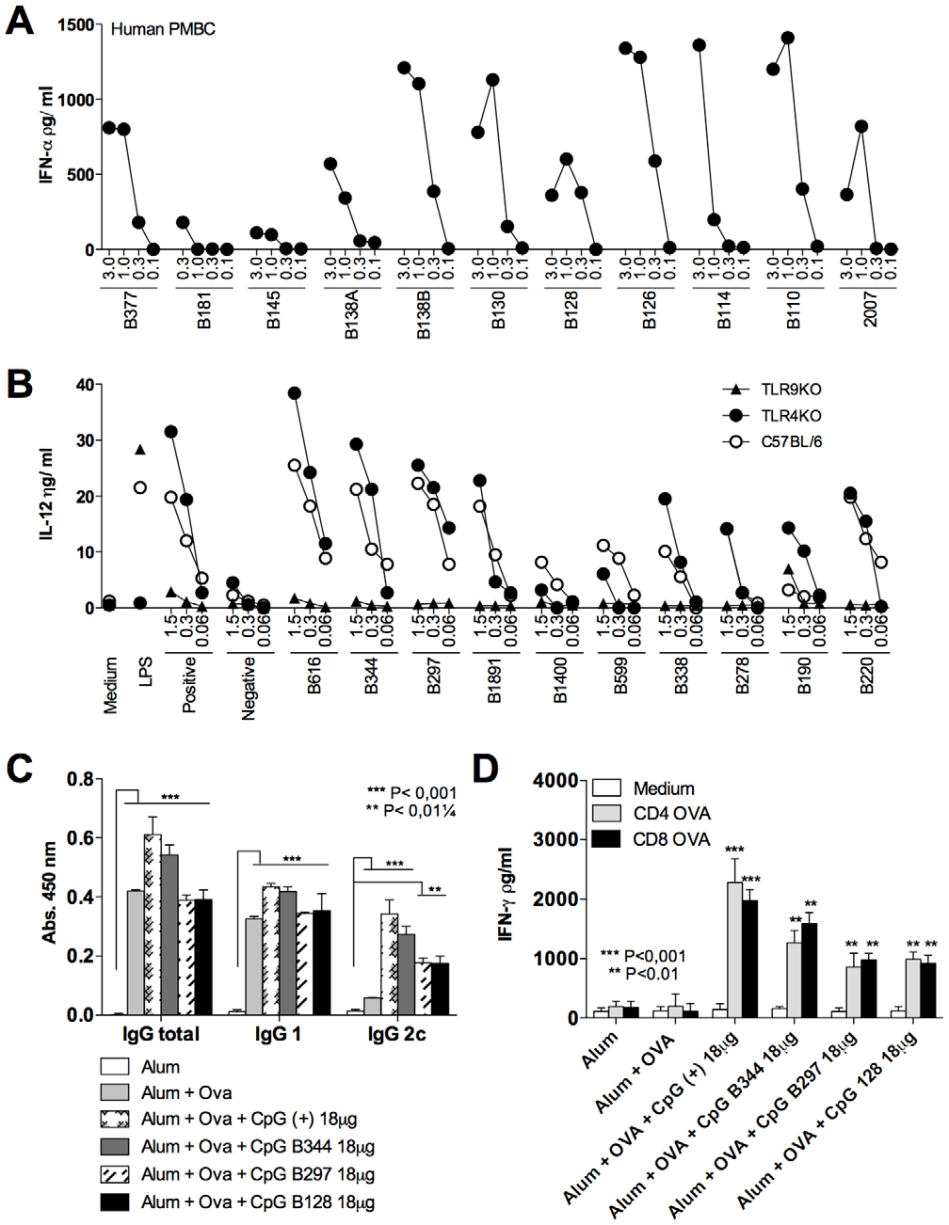
Immunostimulatory and adjuvant activity of TLR9 agonists derived from *T. cruzi* genome. (**A**) PBMCs derived from healthy donors were stimulated with human B-class-like CpG ODNs derived from the *T. cruzi* genome with four different concentrations (3.0, 1.0, 0.3, and 0.1 µM) and the levels of IFN-α measured in the cell culture supernatants 24 h later. The CpG ODN 2007 was used as positive controls for human B-class ODNs. PBMC experiments were performed in three different donors, yielding similar results. (**B**) Proinflammatory activity of mouse B class-like CpG motifs was evaluated in inflammatory macrophages from WT (C57BL/6), *TLR4* ^−/−^ and *TLR9* ^−/−^ mice. ODNs were tested at different concentrations (1.5, 0.3 and 0.06 µM) and LPS, as well as CpG ODN 7909 were used as positive controls for TLR4 and TLR9 activation, respectively. IL-12 (p40) was measured in the macrophage culture supernatants 24 h after cellular stimulation. (**C**) C57BL/6 mice received three immunization doses with alum alone, OVA plus alum or OVA plus alum associated with either CpG ODNs B344, B287, B128 or 7909 (positive control). The levels of OVA-specific total IgG, IgG1 and IgG2c were assessed by ELISA. (**D**) Amount of IFN-γ secreted by splenocytes after stimulation with OVA derived CD4^+^ T or CD8^+^ T cell epitopes was evaluated in culture supernatants 72 hours post-stimulation. Asterisks indicate that differences were statistically significant, when comparing T cell response from mice receiving different vaccine formulations.

As previously reported [22], CpG ODNs derived from *T. cruzi* genome activated human and mouse cells through TLR9 to produce IFN-α and IL-12 respectively (Fig. 2A and 2B). The *T. cruzi* derived CpG motifs B344, B297 and B128 were compared to the bacterial CpG motif (CpG 7909), as a positive control, for their capacity to induce immune response in a vaccine formulation containing OVA as antigen (Fig. 2C and 2D). Similar levels of anti-OVA antibodies were detected in sera from mice immunized with distinct TLR9 agonists, including the IgG2c isotype (Fig. 2C). The IFN-γ production by splenocytes restimulated with OVA-specific peptides demonstrates that all the CpG ODNs were able to induce IFN-γ production by both CD4^+^ T and CD8^+^ T cells (Fig. 2D). The best results were obtained in mice immunized with CpG ODNs 7909 and B344, indicating a potential for the *T. cruzi* derived CpG ODN B344 to be used in vaccine formulations combined with NY-ESO-1.

### Mapping of CD4^+^ T and CD8^+^ T cell epitopes from the cancer/testis antigen NY-ESO-1

**Figure 3:**
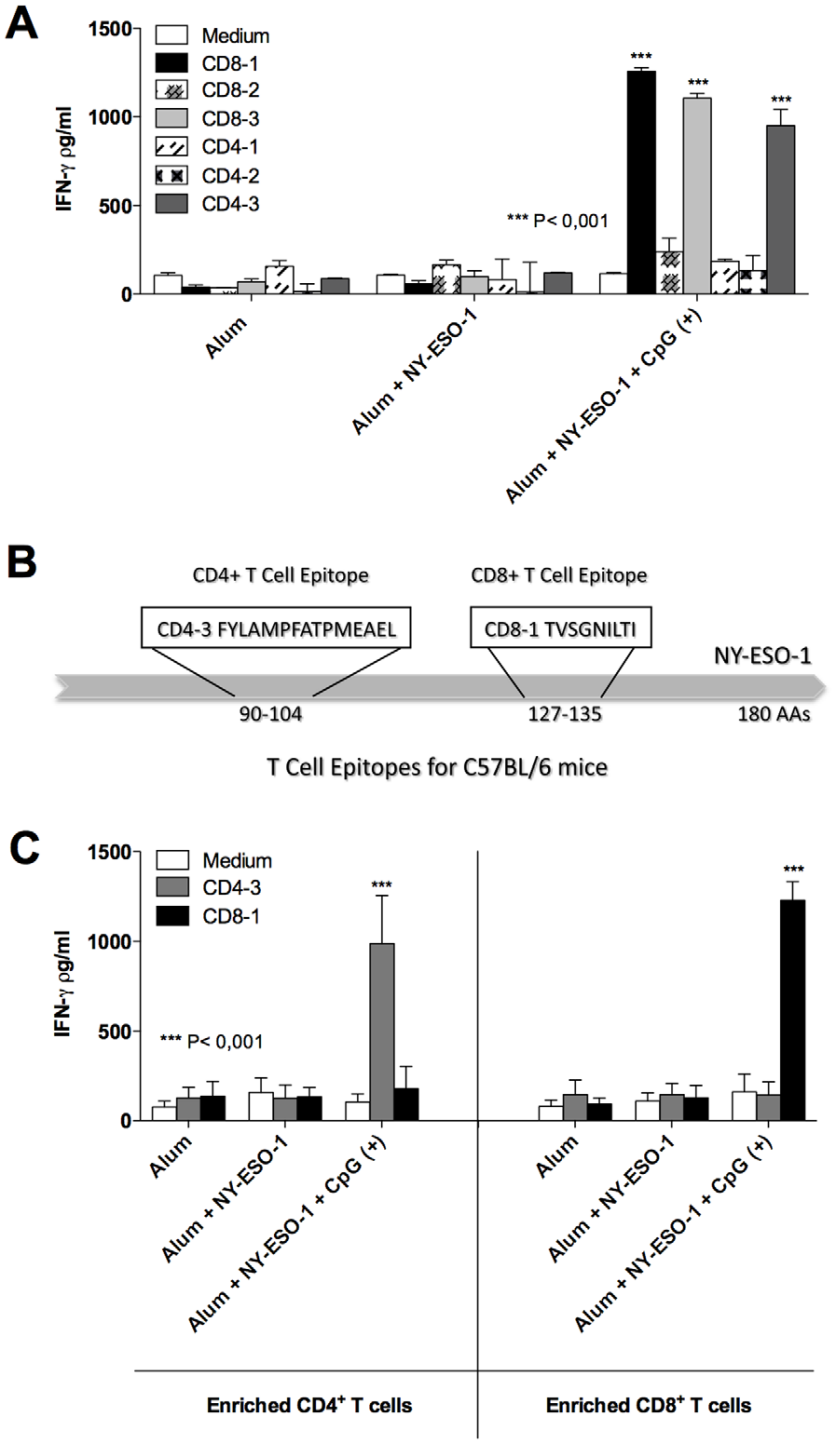
Mapping of immunostimulatory CD4^+^ T and CD8^+^ T cell epitopes present in NY-ESO-1. (**A**) C57BL/6 mice were immunized with alum alone, alum plus rNY-ESO-1 associated or not with CpG ODN 7909 to evaluate the immunostimulatory activity of peptides encoding the putative CD4^+^ T and CD8^+^ T cell epitopes from NY-ESO-1. Mice received three immunization doses at day 0, 14 and 28. Splenocytes were harvested 21 days after the last immunization dose, restimulated *in vitro* with different NY-ESO-1-specific peptides, and the levels of IFN-γ production measured in the cell culture supernatants by ELISA. Asterisks indicate that differences in IFN-γ responses to a specific CD4^+^ T or CD8^+^ T cell peptides (CD8-1, CD8-3 and CD4-3) were statistically significant (p<0.001), when comparing splenocytes from mice receiving the same vaccine formulation, stimulated with CD8-2, CD4-1, CD4-2, or left unstimulated. (**B**) A schematic illustration shows the sequence and position of immunostimulatory CD4^+^ T and CD8^+^ T cell epitopes selected from NY-ESO-1 to be used in this study. (**C**) CD4^+^ T and CD8^+^ T lymphocytes were enriched from total spleen cells of immunized mice by magnetic beads. Each subpopulation was restimulated with CD4-3 and CD8-1 peptides and IFN-γ production evaluated by ELISA after 72 hours incubation.

The ORF of the NY-ESO-1 gene was analyzed for regions that bind to MHC Class I or II, in order to identify NY-ESO-1 specific T cell epitopes. To select the best CD4^+^ T cell epitope and CD8^+^ T cell epitope, three different epitopes for MHC class I and three for MHC class II were tested in an *ex vivo* assay (Figure 3). C57BL/6 mice were immunized with alum alone, alum plus NY-ESO-1, and alum plus NY-ESO-1 plus CpG ODN 7909. The IFN-γ production by NY-ESO-1-specific T cells was detected after restimulation of splenocytes with the different peptides (Fig. 3A). The CD8-1 and CD4-3 epitopes induced higher levels of IFN-γ production and were selected to be used in the next experiments. A schematic illustration shows the localization and sequence of NY-ESO-1 epitopes selected for this study (Fig. 3B). The specificity of selected peptides was confirmed employing highly purified CD4^+^ T or CD8^+^ T cell subsets. Our results show that CD4^+^ T and CD8^+^ T cells produced IFN-γ only when stimulated with peptides CD4-3 or CD8-1, respectively (Fig. 3C).

### A *T. cruzi* derived CpG motif promotes both CD4^+^ T and CD8^+^ T cell responses to NY-ESO-1 and delays development of the B16F10 melanoma cell line expressing NY-ESO-1

**Figure 4:**
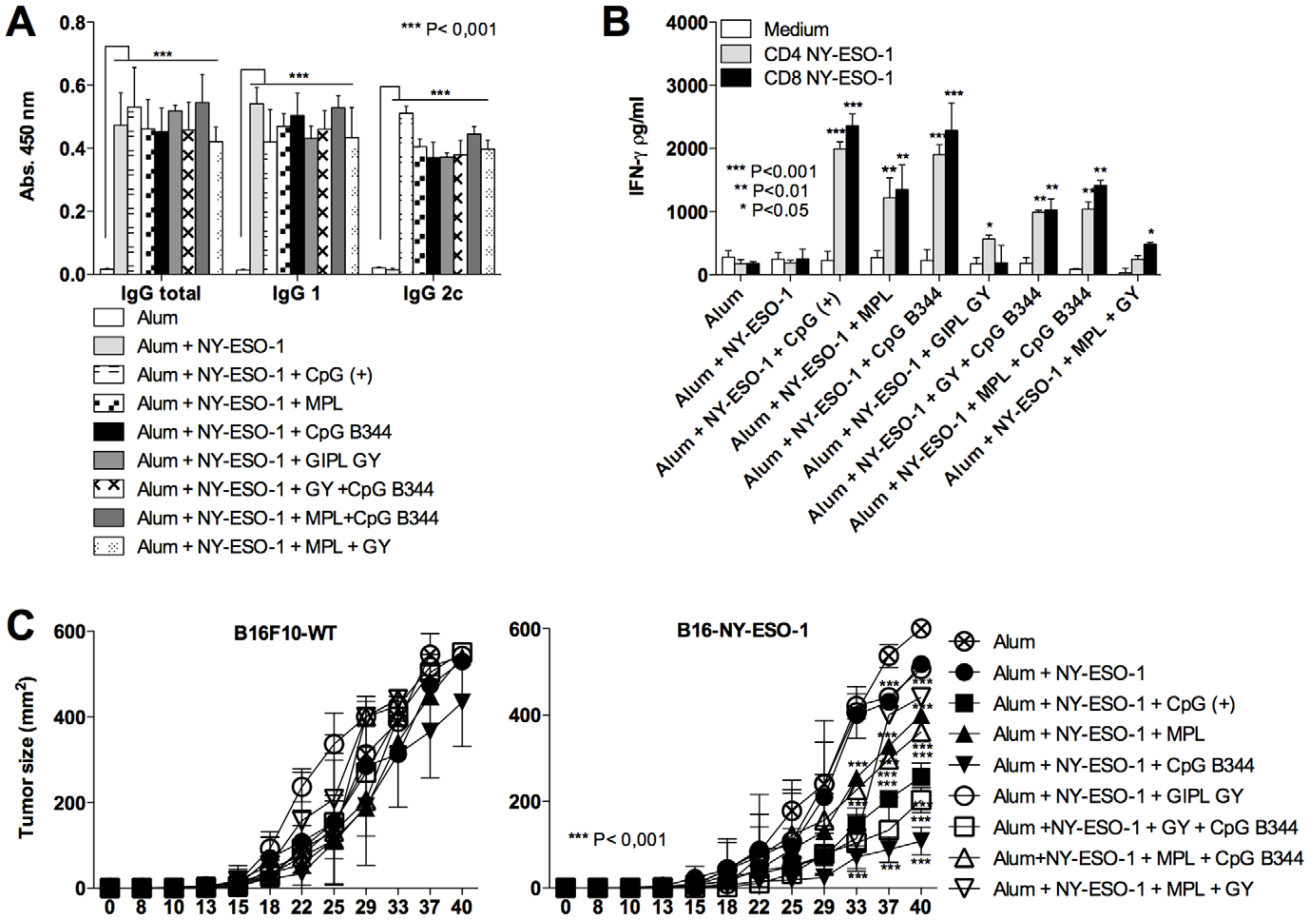
Evaluation of antibody and T cell responses as well as protective immunity elicited by immunization with different formulations containing the tumor-associated NY-ESO-1 antigen. C57BL/6 mice were subjected to three immunization doses on days 0, 14 and 28. (A) Serum levels of NY-ESO-1-specific total IgG, IgG1 and IgG2c; and (B) IFN-γ production by splenocytes stimulated with NY-ESO-1 CD4^+^ T and CD8^+^ T peptides cells were evaluated by ELISA. (C) Control and immunized mice were challenged with 5×10^4^ B16F10 melanoma cell expressing or not NY-ESO-1. The tumor growth was evaluated every 4 days for 40 days after challenge. Asterisks indicate that differences in IFN-γ responses to NY-ESO-1 CD4^+^ T and CD8^+^ T cell peptide and tumor growth are statistically significant, when comparing mice receiving different vaccine formulations.

To test the ability of the *T. cruzi* derived TLR agonists to induce protective immunity to tumor development, we immunized mice with alum plus NY-ESO-1 associated with GIPL GY, CpG ODN B344 or positive and negative controls. The capacity of the vaccine formulations to induce immunological responses anti-NY-ESO-1 and to protect mice against a challenge with B16F10 melanoma cell line expressing NY-ESO-1 was measured (Figure 4). The immunized mice were evaluated for the serum levels of anti-NY-ESO-1 total IgG, IgG1 and IgG2c isotypes. The levels of total IgG, IgG1 and IgG2c were similar when comparing the mice that received different TLR agonists. In contrast, mice that received alum plus NY-ESO-1 without a TLR agonist produced high levels of antigen-specific total IgG and IgG1, but not IgG2c (Fig. 4A). The cellular immune responses were evaluated by measuring the levels of IFN-γ produced by splenocytes from immunized mice after restimulation with NY-ESO-1 derived CD4^+^ T or CD8^+^ T cell epitopes. Our results show that CpG ODN B344 and 7909 (positive control) induced similar levels of IFN-γ (Fig. 4B). Importantly, in mice challenged with B16F10 melanoma expressing NY-ESO-1, the CpG ODN B344 was the most effective TLR agonist in delaying tumor growth. No protection was observed in mice that received the wild type (non-transfected) B16F10 melanoma, indicating that the delay of tumor growth was mediated by NY-ESO-1-specific immune responses (Fig. 4C).

### Treatment with paracetamol alleviates adjuvant-induced hypernociception, but does not affect the protective immunity induced by the NY-ESO-1 vaccine

**Figure 5:**
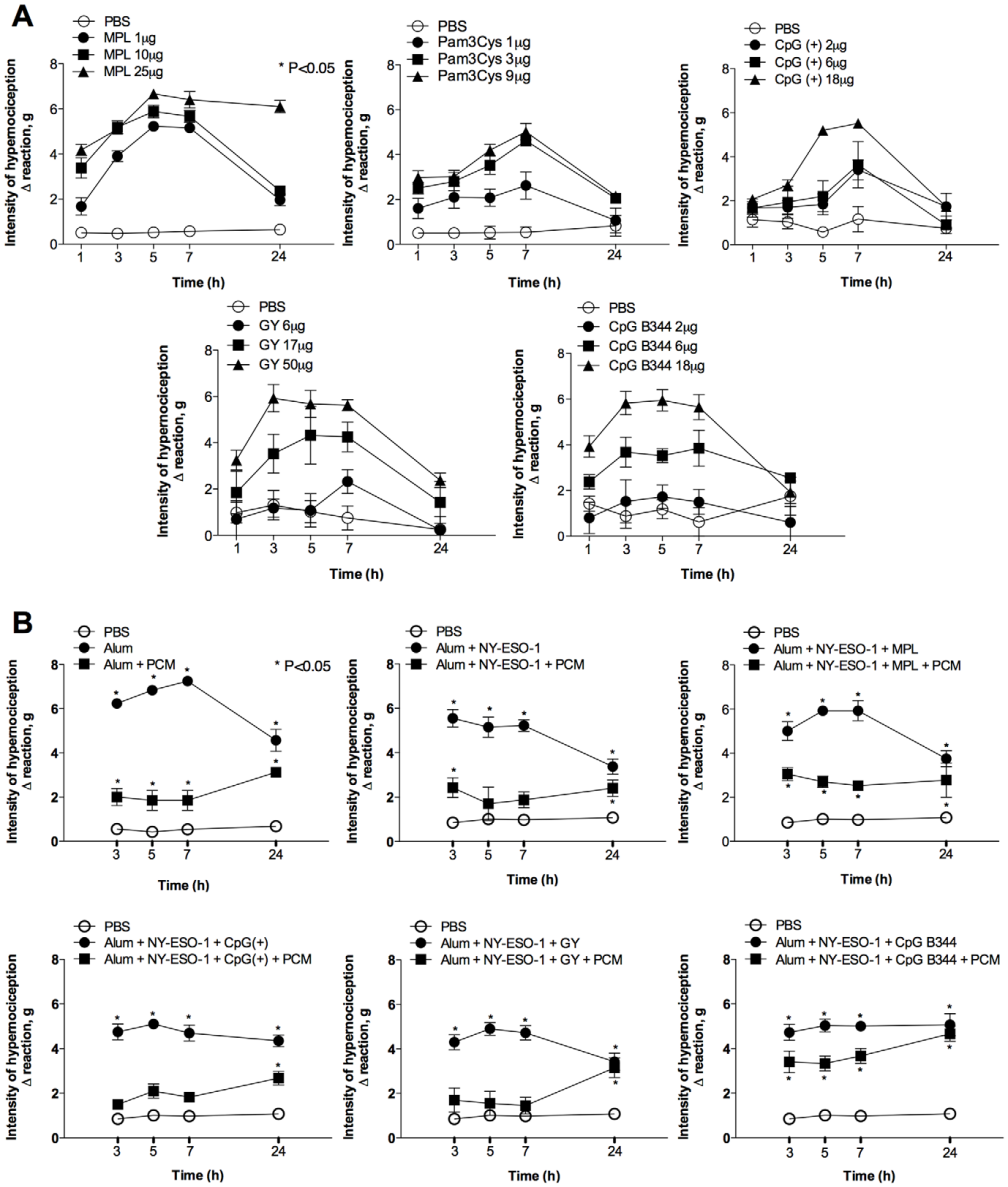
Hypernociception induced by TLR agonists and the NY-ESO-1 vaccine formulations. (**A**) Different TLR agonists were injected in the footpad of mice and hypernociception evaluated at the indicated time points. (**B**) Vaccine formulations containing alum; alum plus NY-ESO-1; or alum plus NY-ESO-1 associated with TLR agonists were given to mice that were left untreated or treated with PCM orally, 30 minutes prior injection with different vaccine formulations. Asterisks mean significant difference when comparing PBS group with TLR agonists experimental groups (*P*<0,05).

**Figure 6:**
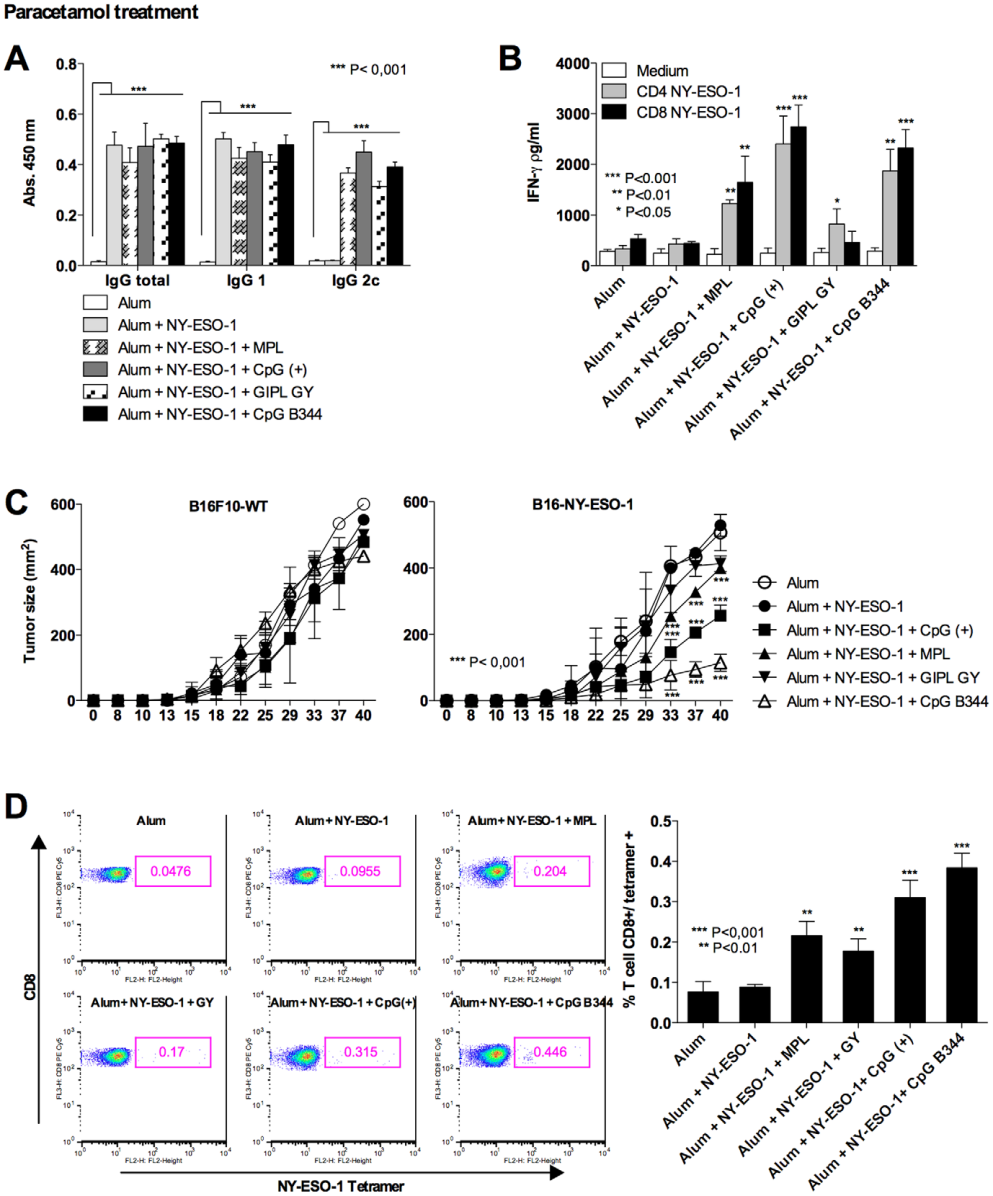
Evaluation of antigen-specific immune response after PCM administration. C57BL/6 mice were subjected to three immunization doses on days 0, 14 and 28. Thirty minutes prior each immunization dose, a group of mice received 10 mg/Kg of PCM by the oral rout. (**A**) Serum levels of NY-ESO-1-specific total IgG, IgG1 and IgG2c; and (**B**) IFN-γ responses by splenocytes stimulated with NY-ESO-1 CD4^+^ T and CD8^+^ T cell peptides were evaluated by ELISA. (**C**) Control and immunized mice were challenged with 5×10^4^ B16F10 melanoma cell expressing or not NY-ESO-1. The tumor growth was evaluated every 4 days for 40 days after challenge. Asterisks indicate that differences in IFN-γ responses to NY-ESO-1 CD4^+^ T and CD8^+^ T cell peptide and curve of tumor growth are statistically significant, when comparing mice receiving different vaccine formulations. (**D**) The frequency of CD8^+^ T cells NY-ESO-1-specific were evaluated by flow cytometry using as marker FITC CD3, PECy5 CD8 and PE NY-ESO-1 tetramer. Are shown on the left representative dot blots of tetramer staining for each experimental group. Graph on the right shows the average (n = 4) of the percentage of CD3^+^/CD8^+^/NY-ESO-1^+^ for each experimental group.

One important aspect of immunological adjuvants is the side effect including the development of inflammation and pain in the site of vaccine injection. In this context, we evaluated whether the immunostimulatory effect of parasite adjuvants and complete vaccine formulation was associated with local hypernociception (decrease in nociceptive threshold). As positive controls we used MPL, Pam3Cys and bacterial CpG motifs. As expected, all the TLR agonists, positive controls or the enquired ones, induced significant increase in hypernociception (Fig. 5A). In a second set of experiments, we used the complete vaccine formulation, including alum and NY-ESO-1 as adjuvant (Fig. 5B). Our results show that alum on its own induced an augmentation of hypernociception, which was not further augmented by the association with specific TLR agonists. Thus, we did not find a correlation with the quality of the immune response (*i.e.*, TCMI and protective immunity) and “pain”. In fact, the presence of alum was the determinant factor for hypernociception in our vaccine formulations. Importantly, PCM partially blocked hypernociception in mice inoculated with our vaccine formulations. Parallel experiments were performed by immunizing mice with the previously described formulations with NY-ESO-1 antigen in association or not with the PCM treatment (Fig. 6). Neither humoral (Fig. 6A) nor cellular (Fig. 6B) immune responses were impaired with the analgesic administration. Importantly, the vaccine-induced delay in tumor development was not affected by treatment with PCM (Fig. 6C). The frequency of antigen-specific CD8^+^ T cells was evaluated by staining splenocytes with anti-CD3, anti-CD8 and NY-ESO-1 tetramer. The frequency of NY-ESO-1-specific CD8^+^ T cells in spleen of vaccinated mice was proportional to the IFN-γ production (Fig. 6D).

## Discussion

Most of the commercially available vaccines that are considered highly effective in eliciting strong and long-lasting protective immunity are thought to be mediated by neutralizing antibodies, as is the case of Tetanus Toxoid, Polyomielitis, Small Pox and Measles vaccines [31], [32]. In contrast, development of efficient vaccines that elicit TCMI, largely responsible for mediating protective immunity to infections such as tuberculosis and leishmaniasis, as well as cancer, is a difficult task [18], [31], [33]. A major challenge for the development of vaccines that induce TCMI, is the establishment of ideal formulations to induce strong and long-lasting protective T cell immunity, mediated by CD8^+^ T lymphocytes [18], [31]. In particular, immunological adjuvants capable of eliciting a strong and long-lived TCMI with the least side effect is a main Achille's heel for development of human vaccines [32].

The finding that activation of innate immune receptors promotes the development of Th1 lymphocytes and TCMI had stimulated the search for new TLR agonists, as potential immunological adjuvants [11], [12]. Our previous studies demonstrated that GPI anchors and GIPLs from *T. cruzi* are able to induce the synthesis of pro-inflammatory cytokines via TLR2 and TLR4, respectively [20], [21]. Furthermore, various CpG ODN sequences derived from *T. cruzi* genome are also able to stimulate cytokine synthesis, including IL-12, both by macrophages and dendritic cells via TLR9 [22]. In the present study, we tested *in vivo* the *T. cruzi* derived Pathogen Associated Molecular Patterns (PAMPs) as immunological adjuvants. We showed that GIPLs from *T. cruzi* are potent inducers of antigen-specific immune responses, as measured by IFN-γ production by CD4^+^ lymphocytes as well as serum levels of IgG2c. Furthermore, CpG ODNs derived from *T. cruzi*, in especial the B344 induced an antigen-specific immune response by CD8^+^ T lymphocytes, leading to a delay in tumor development in an antigen-specific manner.

Acquired immunity to tumors involves both humoral and cellular compartments. While antigen-specific antibodies have been demonstrated to mediate protection [34], CD8^+^ T lymphocytes are thought to be the main effector cells, which mediate cytotoxic activity against tumor cells [35]. In addition, both CD8^+^ T as well as CD4^+^ T lymphocytes are important sources of IFN-γ that has various roles in inducing effector mechanisms thought to mediate antitumor activities, which includes activation of effector functions by macrophages [36]. Thus, a critical step for developing effective vaccines is to learn how to induce the appropriate immune response necessary to control tumor growth. Different strategies have been employed to induce strong T cell-mediated immunity and in particular CD8^+^ T cells specific for tumor antigens. These strategies include the use of plasmids (naked DNA) [37] as well as live attenuated viral and bacterial vectors, such as adenovirus [38], MVA [39], *Salmonella sp*. [6] and *Listeria monocytogenis* [40], respectively. In addition, the discovery that Toll-like receptors are activated by viral and bacterial products, also named PAMPs (*e.g.*, LPS, DNA, RNA, lipopeptides, flagelin, Poli-IC) as well as synthetic components (e.g. imiquimod, CpG ODN, Pam3Cys) [18] with proinflammatory and immunologic activity has boosted the field of vaccine development [11].

Briefly, cells from the innate immune system exposed to microbial/synthetic products, synthesize high levels of pro-inflammatory cytokines, such as IL-12 and TNF-α, that are responsible for initiation of IFN-γ synthesis by natural killer cells [41], [42]. In addition, when DCs are exposed to certain PAMPs they express co-receptors, in order to initiate the activation process of naïve T cells, making a bridge between the innate and acquired immunity [11], [14], [18], [41], [42], [43]. Furthermore, DCs activated with TLR agonists will produce IL-12, which will influence the differentiation of CD4^+^ T cells into the Th1 lymphocytes, a main source of IFN-γ, and orchestrate the establishment of TCMI. IFN-γ is critical for class switch of Ig isotypes [44] and activation of effector mechanisms displayed by macrophages that are often involved in host resistance to tumors [35], [36]. Moreover, activation of antigen presenting cells favors cross-presentation, allowing presentation of exogenous antigens via MHC class I and favoring the development of antigen-specific CD8^+^ T cells [17]. Of note, vaccine formulations containing TLR agonists associated with self-antigens, often result in break of tolerance and development of strong immune response to the employed self-antigen [45]. The vaccine formulation employing CT antigens, including NY-ESO-1 and TLR agonists as immunological adjuvants are being widely tested in pre-clinical and clinical trials, both in prophylactic and therapeutic vaccines to fight cancer cells [15], [18]. Some of these formulations also employ combinations of different adjuvants aiming at the induction of a more robust immune response against the tumor antigens [6], [46], [47].

Intracellular protozoan parasites, such as *Toxoplasma gondii*, *T. cruzi* as well as *Leishmania major* are known to induce a strong and long lasting TCMI, which is characterized by highly polarized Th1 lymphocytes and strong CD8^+^ T cell responses [48], [49]. The induction of Th1 lymphocytes and TCMI during protozoan infection is highly dependent of IL-12 [50] as well as Myeloid differentiation primary response gene (88) (MyD88) and TLRs [49], [51]. Studies performed in our laboratory have dedicated to indentify parasite PAMPs that are critical for activating TLRs and initiating the IL-12 production and TCMI during protozoan infections [49]. We identified and characterized the structure of GPI anchors derived from *T. cruzi* as agonists of TLR2 and TLR4 [20], [21]. The lipid moiety of GPI anchors and GIPLs from *T. cruzi* was shown to be an essential moiety that determines the pro-inflammatory activity of these glycolipids, both in rodent and human cells [20], [21], [52], [53]. In more recent studies, we determined that both DNA and RNA activate, respectively, TLR9 and TLR7 and are critical parasite components for induction of IL-12 and host resistance to a primary infection with *T. cruzi* [22], [54], [55]. Furthermore, we identified multiple CpG motifs that activate either mouse or human TLR9 [22]. While the CpG motifs with optimal immunostimulatory activity in mouse and human cells are distinct, the results presented here, employing a CpG motif for mouse, indicate the potential use of the CpG ODNs for human vaccines.

One of the main impediments for approval of new immunological adjuvants for vaccine formulations is their side effect. Due to their intrinsic pro-inflammatory activity, immunological adjuvants often induce undesirable effects, for instance, local edema, rubor and pain, and even systemic effects, such as headache and fever [56], [57], [58]. Here, we addressed this question by testing hypernociception, a measure of “pain”, as a consequence of inflammation elicited by our vaccine formulations. Whereas, each of the TLR agonists used in our formulations induced hypernociception on its own, the alum, an immunological adjuvant approved for use in human vaccines, was sufficient to induce the maximal score of “pain”. Furthemore, addition of specific TLR agonists did not augment the hypernociception scores induced by alum. Importantly, treatment with PCM, which acts at least in part through inhibition of prostaglandin release [59], alleviated the intensity of hypernociception without affecting the quality of the immune responses elicited by the vaccine formulations containing the different parasite adjuvants.

In conclusion, our experiments employing vaccine formulations containing NY-ESO-1 demonstrate that *T. cruzi* derived TLR agonists, *e.g.* GIPLs and CpG ODNs, are efficient immunological adjuvants. Importantly, the use of *T. cruzi* derived CpG ODNs as immunological adjuvant in our vaccine formulation resulted in a significant delay in the growth of the B16F10 melanoma cell line expressing NY-ESO-1. The protective immunity correlated with the magnitude of CD8^+^ T cell response induced by a specific TLR agonist. Finally, our results show that in our vaccine formulations alum was the main component inducing hypernociception, which was alleviated by the administration of PCM. Thus, parasite adjuvants should be further explored in the development of vaccine formulations, aiming to induce both humoral and cellular-mediated immune responses.

## References

[1] Scanlan MJ, Gure AO, Jungbluth AA, Old LJ, Chen YT (2002). Cancer/testis antigens: an expanding family of targets for cancer immunotherapy.. Immunol Rev.

[2] Scanlan MJ, Simpson AJ, Old LJ (2004). The cancer/testis genes: review, standardization, and commentary.. Cancer Immun.

[3] Wang RF, Johnston SL, Zeng G, Topalian SL, Schwartzentruber DJ (1998). A breast and melanoma-shared tumor antigen: T cell responses to antigenic peptides translated from different open reading frames.. J Immunol.

[4] Zarour HM, Maillere B, Brusic V, Coval K, Williams E (2002). NY-ESO-1 119–143 is a promiscuous major histocompatibility complex class II T-helper epitope recognized by Th1- and Th2-type tumor-reactive CD4+ T cells.. Cancer Res.

[5] Jager E, Chen YT, Drijfhout JW, Karbach J, Ringhoffer M (1998). Simultaneous humoral and cellular immune response against cancer-testis antigen NY-ESO-1: definition of human histocompatibility leukocyte antigen (HLA)-A2-binding peptide epitopes.. J Exp Med.

[6] Nishikawa H, Sato E, Briones G, Chen LM, Matsuo M (2006). In vivo antigen delivery by a Salmonella typhimurium type III secretion system for therapeutic cancer vaccines.. J Clin Invest.

[7] Aoki M, Ueda S, Nishikawa H, Kitano S, Hirayama M (2009). Antibody responses against NY-ESO-1 and HER2 antigens in patients vaccinated with combinations of cholesteryl pullulan (CHP)-NY-ESO-1 and CHP-HER2 with OK-432.. Vaccine.

[8] Nishikawa H, Tsuji T, Jager E, Briones G, Ritter G (2008). Induction of regulatory T cell-resistant helper CD4+ T cells by bacterial vector.. Blood.

[9] Gnjatic S, Altorki NK, Tang DN, Tu SM, Kundra V (2009). NY-ESO-1 DNA vaccine induces T-cell responses that are suppressed by regulatory T cells.. Clin Cancer Res.

[10] Kawai T, Akira S (2010). The role of pattern-recognition receptors in innate immunity: update on Toll-like receptors.. Nat Immunol.

[11] Takeuchi O, Akira S (2010). Pattern recognition receptors and inflammation.. Cell.

[12] Iwasaki A, Medzhitov R (2010). Regulation of adaptive immunity by the innate immune system.. Science.

[13] Mosmann TR, Coffman RL (1989). TH1 and TH2 cells: different patterns of lymphokine secretion lead to different functional properties.. Annu Rev Immunol.

[14] Sallusto F, Lanzavecchia A, Araki K, Ahmed R (2010). From vaccines to memory and back.. Immunity.

[15] Adams S (2009). Toll-like receptor agonists in cancer therapy.. Immunotherapy.

[16] Melief CJ, Van Der Burg SH, Toes RE, Ossendorp F, Offringa R (2002). Effective therapeutic anticancer vaccines based on precision guiding of cytolytic T lymphocytes.. Immunol Rev.

[17] Heath WR, Carbone FR (2009). Dendritic cell subsets in primary and secondary T cell responses at body surfaces.. Nat Immunol.

[18] Coffman RL, Sher A, Seder RA (2010). Vaccine adjuvants: putting innate immunity to work.. Immunity.

[19] Krieg AM (2008). Toll-like receptor 9 (TLR9) agonists in the treatment of cancer.. Oncogene.

[20] Campos MA, Almeida IC, Takeuchi O, Akira S, Valente EP (2001). Activation of Toll-like receptor-2 by glycosylphosphatidylinositol anchors from a protozoan parasite.. J Immunol.

[21] Oliveira AC, Peixoto JR, de Arruda LB, Campos MA, Gazzinelli RT (2004). Expression of functional TLR4 confers proinflammatory responsiveness to Trypanosoma cruzi glycoinositolphospholipids and higher resistance to infection with T. cruzi.. J Immunol.

[22] Bartholomeu DC, Ropert C, Melo MB, Parroche P, Junqueira CF (2008). Recruitment and endo-lysosomal activation of TLR9 in dendritic cells infected with Trypanosoma cruzi.. J Immunol.

[23] Lien E, Sellati TJ, Yoshimura A, Flo TH, Rawadi G (1999). Toll-like receptor 2 functions as a pattern recognition receptor for diverse bacterial products.. J Biol Chem.

[24] Maraskovsky E, Sjolander S, Drane DP, Schnurr M, Le TT (2004). NY-ESO-1 protein formulated in ISCOMATRIX adjuvant is a potent anticancer vaccine inducing both humoral and CD8+ t-cell-mediated immunity and protection against NY-ESO-1+ tumors.. Clin Cancer Res.

[25] Aida Y, Pabst MJ (1990). Removal of endotoxin from protein solutions by phase separation using Triton X-114.. J Immunol Methods.

[26] Parker KC, Bednarek MA, Coligan JE (1994). Scheme for ranking potential HLA-A2 binding peptides based on independent binding of individual peptide side-chains.. J Immunol.

[27] Rammensee H, Bachmann J, Emmerich NP, Bachor OA, Stevanovic S (1999). SYFPEITHI: database for MHC ligands and peptide motifs.. Immunogenetics.

[28] Previato JO, Gorin PA, Mazurek M, Xavier MT, Fournet B (1990). Primary structure of the oligosaccharide chain of lipopeptidophosphoglycan of epimastigote forms of Trypanosoma cruzi.. J Biol Chem.

[29] Parada CA, Vivancos GG, Tambeli CH, Cunha FQ, Ferreira SH (2003). Activation of presynaptic NMDA receptors coupled to NaV1.8-resistant sodium channel C-fibers causes retrograde mechanical nociceptor sensitization.. Proc Natl Acad Sci U S A.

[30] Cunha TM, Verri WA, Vivancos GG, Moreira IF, Reis S (2004). An electronic pressure-meter nociception paw test for mice.. Braz J Med Biol Res.

[31] Zinkernagel RM, Hengartner H (2006). Protective ‘immunity’ by pre-existent neutralizing antibody titers and preactivated T cells but not by so-called ‘immunological memory’.. Immunol Rev.

[32] Plotkin SA (2010). Correlates of protection induced by vaccination.. Clin Vaccine Immunol.

[33] Kaufmann SH (2010). Future vaccination strategies against tuberculosis: thinking outside the box.. Immunity.

[34] Valmori D, Souleimanian NE, Tosello V, Bhardwaj N, Adams S (2007). Vaccination with NY-ESO-1 protein and CpG in Montanide induces integrated antibody/Th1 responses and CD8 T cells through cross-priming.. Proc Natl Acad Sci U S A.

[35] Dunn GP, Bruce AT, Ikeda H, Old LJ, Schreiber RD (2002). Cancer immunoediting: from immunosurveillance to tumor escape.. Nat Immunol.

[36] Miller CH, Maher SG, Young HA (2009). Clinical Use of Interferon-gamma.. Ann N Y Acad Sci.

[37] Donnelly JJ, Wahren B, Liu MA (2005). DNA vaccines: progress and challenges.. J Immunol.

[38] Chen PW, Wang M, Bronte V, Zhai Y, Rosenberg SA (1996). Therapeutic antitumor response after immunization with a recombinant adenovirus encoding a model tumor-associated antigen.. J Immunol.

[39] Carroll MW, Overwijk WW, Chamberlain RS, Rosenberg SA, Moss B (1997). Highly attenuated modified vaccinia virus Ankara (MVA) as an effective recombinant vector: a murine tumor model.. Vaccine.

[40] Singh R, Wallecha A (2011). Cancer immunotherapy using recombinant Listeria monocytogenes: Transition from bench to clinic.. Hum Vaccin.

[41] Trinchieri G (2003). Interleukin-12 and the regulation of innate resistance and adaptive immunity.. Nat Rev Immunol.

[42] Seder RA, Gazzinelli R, Sher A, Paul WE (1993). Interleukin 12 acts directly on CD4+ T cells to enhance priming for interferon gamma production and diminishes interleukin 4 inhibition of such priming.. Proc Natl Acad Sci U S A.

[43] Kis-Toth K, Szanto A, Thai TH, Tsokos GC (2011). Cytosolic DNA-Activated Human Dendritic Cells Are Potent Activators of the Adaptive Immune Response.. J Immunol.

[44] Rakoff-Nahoum S, Medzhitov R (2009). Toll-like receptors and cancer.. Nature Reviews Cancer.

[45] Yang Y, Huang CT, Huang X, Pardoll DM (2004). Persistent Toll-like receptor signals are required for reversal of regulatory T cell-mediated CD8 tolerance.. Nat Immunol.

[46] Zaks K, Jordan M, Guth A, Sellins K, Kedl R (2006). Efficient immunization and cross-priming by vaccine adjuvants containing TLR3 or TLR9 agonists complexed to cationic liposomes.. J Immunol.

[47] Kasturi SP, Skountzou I, Albrecht RA, Koutsonanos D, Hua T (2011). Programming the magnitude and persistence of antibody responses with innate immunity.. Nature.

[48] Gazzinelli RT, Ropert C, Campos MA (2004). Role of the Toll/interleukin-1 receptor signaling pathway in host resistance and pathogenesis during infection with protozoan parasites.. Immunol Rev.

[49] Gazzinelli RT, Denkers EY (2006). Protozoan encounters with Toll-like receptor signalling pathways: implications for host parasitism.. Nat Rev Immunol.

[50] Aliberti JC, Cardoso MA, Martins GA, Gazzinelli RT, Vieira LQ (1996). Interleukin-12 mediates resistance to Trypanosoma cruzi in mice and is produced by murine macrophages in response to live trypomastigotes.. Infect Immun.

[51] Campos MA, Closel M, Valente EP, Cardoso JE, Akira S (2004). Impaired production of proinflammatory cytokines and host resistance to acute infection with Trypanosoma cruzi in mice lacking functional myeloid differentiation factor 88.. J Immunol.

[52] Camargo MM, Almeida IC, Pereira ME, Ferguson MA, Travassos LR (1997). Glycosylphosphatidylinositol-anchored mucin-like glycoproteins isolated from Trypanosoma cruzi trypomastigotes initiate the synthesis of proinflammatory cytokines by macrophages.. J Immunol.

[53] Almeida IC, Camargo MM, Procopio DO, Silva LS, Mehlert A (2000). Highly purified glycosylphosphatidylinositols from Trypanosoma cruzi are potent proinflammatory agents.. Embo J.

[54] Caetano BC, Carmo BB, Melo MB, Cerny A, dos Santos SL (2011). Requirement of UNC93B1 reveals a critical role for TLR7 in host resistance to primary infection with Trypanosoma cruzi.. J Immunol.

[55] Bafica A, Santiago HC, Goldszmid R, Ropert C, Gazzinelli RT (2006). Cutting edge: TLR9 and TLR2 signaling together account for MyD88-dependent control of parasitemia in Trypanosoma cruzi infection.. J Immunol.

[56] Su L, Tucker R, Frey SE, Gress JO, Chan IS (2000). Measuring injection-site pain associated with vaccine administration in adults: a randomised, double-blind, placebo-controlled clinical trial.. J Epidemiol Biostat.

[57] Taddio A, Ilersich AL, Ipp M, Kikuta A, Shah V (2009). Physical interventions and injection techniques for reducing injection pain during routine childhood immunizations: systematic review of randomized controlled trials and quasi-randomized controlled trials.. Clin Ther.

[58] Shah V, Taddio A, Rieder MJ (2009). Effectiveness and tolerability of pharmacologic and combined interventions for reducing injection pain during routine childhood immunizations: systematic review and meta-analyses.. Clin Ther.

[59] Anderson BJ (2008). Paracetamol (Acetaminophen): mechanisms of action.. Paediatr Anaesth.

